# Contribution of Direct Cerebral Vascular Transport in Brain Substance Clearance

**DOI:** 10.14336/AD.2023.0426

**Published:** 2024-04-01

**Authors:** Qiuting Wen, Haoyu Wang, E. Mark Haacke, Quan Jiang, Jiani Hu

**Affiliations:** ^1^Department of Radiology and Imaging Sciences, Indiana University, Indianapolis, IN, USA.; ^2^Beijing Institute of Radiation Medicine, Beijing, China.; ^3^Department of Radiology, Wayne State University, Detroit, MI 48201 USA.; ^4^Department of Neurology, Henry Ford Health System, Detroit, MI 48202 USA.

**Keywords:** substance clearance, vascular circulation, CSF, glymphatic system

## Abstract

The accumulation of harmful substances has long been recognized as a likely cause of many neurodegenerative diseases. The two classic brain clearance pathways are cerebrospinal fluid (CSF) and vascular circulation systems. Since the discovery of the glymphatic system, research on the CSF pathway has gained momentum, and impaired CSF clearance has been implicated in virtually all neurodegenerative animal models. However, the contribution of the direct participation of vascular transport across the blood-brain barrier in clearing substances is often ignored in glymphatic papers. Supportive evidence for the direct involvement of parenchymal vasculature in substance clearance is accumulated. First, multiple mechanisms have been proposed for the vascular drainage of exogenous and endogenous substances across the blood-brain barriers. Second, the "traditional" role of arachnoid villi and granulations as the main site for CSF draining into the vasculature system has been questioned. Third, MRI studies using different CSF tracers indicate that parenchymal vasculature directly participates in tracer efflux, consistent with immunohistochemical findings. Here we will review evidence in the literature that supports the direct participation of the parenchymal vascular system in substance clearance, in addition to the CSF clearance pathways.

## 1. Introduction

Harmful substance removal from the brain is essential for maintaining brain homeostasis across the lifespan [1]. A critical function of sleep is to remove harmful substances more effectively [2]. The accumulation of harmful substances, including heavy metals and waste proteins, has long been hypothesized to cause many neuro-degenerative diseases. For instance, Alzheimer's disease (AD) is marked by the accumulation of protein aggregates in the brain parenchyma, including amyloid-β (Aβ) plaques and hyperphosphorylated tau tangles. The imbalance between protein production and clearance has been increasingly recognized as a pathway to the pathogenesis of AD, which starts long before the symptom onsite [3-12]. It is, therefore, critical to understand how harmful substances are cleared from the brain parenchyma and how this process alterates in neurodegenerative diseases.

**Figure 1.**
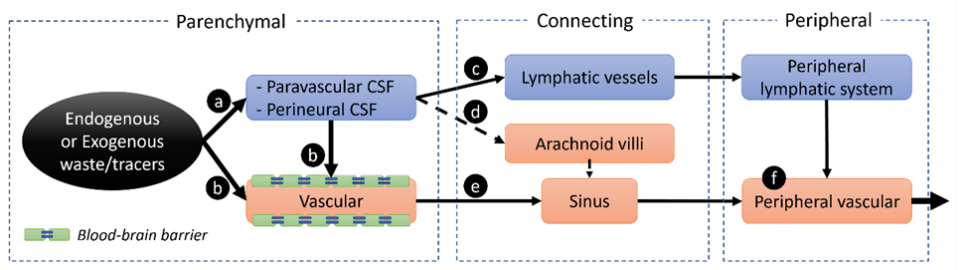
**Summary of possible efflux routes of interstitial substances (endogenous or exogenous waste products) into the peripheral vascular system**. Inside the parenchyma, the endogenous or exogenous substances can efflux either into the paravascular/perineural CSF (a), or into the vascular blood across the blood-brain barrier (b) to rapidly circulate into the peripheral vasculature (eγf). For those drained through the paravascular/perineural CSF (a), it will travel along this space to exit the parenchyma and transport either into connecting lymphatic vessels (e.g., meningeal lymphatic vessels, nasal mucosa, etc.) (c), or into the dura sinus through arachnoid villi/granulations (d). Eventually, all substance will drain into the peripheral vascular system (f).

The brain's anatomy is unique: the parenchyma and all its communication channels with the outside are completely encased in cerebrospinal fluid (CSF) [13], including the parenchyma itself, all 12 pairs of cranial nerves, all vascular-communication channels, and the spinal cord. There are several consequences associated with this unique anatomy. First, it indicates that there are only two physical pathways for parenchymal waste removal: the CSF circulation pathways (Fig. 1a) and the parenchymal vascular pathways (Fig. 1b). Second, it suggests a dual mission of the brain vessels: blood transportation and CSF pumping. The reliance of CSF circulation on pulsation and vasomotion has been shown in both animal and human studies [14-19]. Third, material exchange between the brain parenchyma and the lymphatic system can only happen through the CSF because all brain's communications to the outside are bathed in CSF, including those in paravascular spaces surrounding vascular-communication channels and those in peri-neural spaces surrounding 12 pairs of cranial nerves. This suggests that CSF plays a critical role in regulating brain immunity. Fourth, substance removal from brain parenchyma can be studied with the CSF administration of tracers without direct injection into the brain tissues.

The traditional view of parenchymal tracers getting into the blood pool is focused on arachnoid villi and granulations [20-22] (Fig. 1d). However, this view has been questioned by accumulating data from in vivo and post-mortem studies [23-25]. If arachnoid villi and granulations are not the main outflow pathway of CSF draining to the vascular system [25], which pathway contributes to the rapid observation of CSF tracer in the peripheral vascular system? Recent MRI results have demonstrated evident participation of the parenchymal venous system in draining the CSF tracers following intra-cisterna magna infusion of different MRI tracers [26]. This is consistent with the results of detecting CSF tracers in cerebral vascular walls by immunohistochemistry [25]. Moreover, various vascular transport mechanisms, including ATP binding cassette (ABC) transporters, solute carrier (SLC), and receptor mediated transcytosis (RMT), have been identified for cerebral clearance of both exogenous and endogenous substances across the blood-brain barrier, further strengthening the participation of parenchymal vascular system in substance clearance. Below, we will review evidence from different perspectives that jointly support an unignorable role of the parenchymal vascular system in waste clearance, aiming to raise awareness of the vasculature route when studying CSF pathways.

## 2. The consequence of the unique anatomy of the brain parenchyma

Brain parenchyma is the most crucial organ in the human body. In addition to protect it from physical insults, the brain's anatomy must also meet its demands for high energy consumption (3-5% of body weight but consumption of 15-25% of energy) and the corresponding requirement for effective and timely waste removal.

Unlike most peripheral vessels where arterial vessels are paired with venous vessels nearby, arterial vessels are separated from venous vessels inside the brain. This can be considered the anatomical basis for the glymphatic system, in that such a separation facilitates the interstitial fluid drainage into the venous side through the para-arterial to the para-venous flow gradient. In addition to the unique wiring of the vasculature, the brain parenchyma is also completely bathed in CSF: not only the brain parenchyma itself is surrounded by CSF, all its communication channels to the outside are also enclosed by CSF. Specifically, all parenchymal arterial and venous communication channels are surrounded by CSF-filled para-arterial space and para-venous space. The para-arterial and para-venous spaces are also termed influx and efflux pathways in the glymphatic theory, respectively. Similarly, all parenchymal nerve communication channels to the outside, including 12 pairs of cranial nerves and the spinal cord, are also surrounded by CSF-filled perineural space [27]. As a result, waste substances must exit through the CSF pathways (Fig. 1a) and/or through parenchymal vasculature by crossing BBB (Fig. 1b). All substances in these two pathways will eventually drain toward the peripheral vascular system (Fig. 1f).

Such CSF-encapsulated brain's anatomy suggests that there are possibly three main efflux routes by which the interstitial substances can reach the peripheral vascular system: 1) directly transport across BBB into the vascular system within the parenchyma (Fig. 1bγe); 2) travel along the paravascular space to exit the parenchyma and then enter the sinus system through arachnoid villi and granulations (Fig. 1aγd), 3) travel along the paravascular space to exit the parenchyma and then enter the connecting lymphatic vessels which innervate the para-brain area, then into the peripheral lymphatic system (Fig. 1aγc). Anatomically, the connecting lymphatic vessels, including the nasal lymphatics and the recently discovered meningeal lymphatic vessels at the skull base, are outside the brain parenchyma (Fig. 1c). Among perineural drainage pathways, the most studied ones are along the olfactory nerve (CN I), followed by the optic (CN II) nerve, and the trigeminal nerve (CN V). Other less-studied cranial nerve pathways include the glossopharyngeal (CN IX), vagus (CN X), accessory (CN XI) nerves, and facial nerve (CN VII). The unstudied CN III, IV, and VI theoretically may also participate in parenchymal substance clearance. Because the circulation speed of CSF is at least one order slower than that of blood, it would be desirable for the substances to enter the vascular system inside the parenchyma for fast drainage. For these reasons, it is crucial to determine whether the parenchymal vascular system directly participates in substance clearance.

Another consequence of the unique anatomy of the brain parenchyma is that the interaction between the brain parenchyma and the lymphatic system must involve the CSF clearance system, because all communication channels connecting parenchyma to the outside are surrounded by CSF and the brain parenchyma is devoid of lymph. For this reason, the paravascular space is expected to play a crucial role in the immunity of the brain parenchyma, which is consistent with the clinical manifestation of enlarged paravascular space in many neurological diseases [28-31].

Interestingly, the brain's CSF circulation network also offers unique opportunities to study the waste clearance pathways through CSF tracer injection without injuring the parenchymal tissue. Keep parenchymal tissue intact is key to understanding the brain clearance function under normal physiology, as parenchymal clearance is sensitive to many physiological parameters, including blood pressure [7], sleep [32], anesthesia [33-35], body posture [36], and aging [4, 7, 37-43]. For example, a 10 bpm decrease in heart rate (bradycardia) reduces substance clearance and results in a nearly 20% additional accumulation of Aβ in the brain parenchyma, whereas a 30 bpm increase in heart rate (tachycardia) showed a nearly 30% reduction in Aβ levels in the brain parenchyma [7]. Thus, any large perturbation and invasive procedures (i.e., direct injection into the parenchymal tissue) may alter the glymphatic function and, therefore, should be avoided. Fortunately, the unique design of the CSF network allows the intra-cisterna magna infused tracers to be delivered to the brain parenchyma without disturbing brain tissue. This injection approach has become a standard practice for tracking waste clearance pathways and investigating clearance functions in both animal and human studies [44].

## 3. The debated role of arachnoid villi and granulations in CSF drainage

Arachnoid villi or granulations have long been considered a primary site for CSF circulating back to the bloodstream through the dura sinus [45, 46]. As a result, it has been believed that the vascular participation of parenchymal waste clearance mainly occurs at the dural sinus level through arachnoid villi/granulations that project into the dural venous sinuses. Thus, the current paradigm suggests a dual-outflow system for CSF to reach the blood circulation, one directly to the venous blood through arachnoid projections (Fig. 1aγd) and one indirectly through the lymphatic system (Fig. 1aγc). However, the view of arachnoid villi/granulations as a major drainage site is debated [47, 48] and has been challenged by results from recent animal and human studies.

Recent mice studies have suggested that the arachnoid villi-sinus pathway plays a minimum role in draining the CSF-injected tracers [49, 50]. The studies reported *non-*detectible tracer outflow in the level of dural sinuses for both large and small molecular tracers, with the majority outflow through perineural routes to the lymphatic system. Their observations were also consistent with earlier MRI studies in dogs and pigs where tracers injected into the cisterna magna were observed across the cribriform plate, nasal (sub)mucosa, and periorbital regions, but no enhancement was observed in proximity to the superior sagittal sinus [51-53]. These findings collectively challenged the traditional view of arachnoid villi as a major outflow route of solute from CSF. In fact, historical data has never reached a consensus on the relative contribution of the arachnoid pathway to drain CSF tracers. In some species, such as rabbits and sheep, lymphatic vessels accounted for around 30-50% of total outflow. The remainder was *assumed* to be drained through arachnoid villi without direct evidence [54, 55]. The mice studies questioned this assumption and suggested that arachnoid villi are, at most, a secondary pathway for CSF drainage.

A recent human study with a large number of post-mortem patient samples and high-quality of histological and MRI data provided extensive details on the anatomy, morphology, and cytologic composition of arachnoid granulations, which suggested that the main role of arachnoid granulations serve as immune hubs at meningeal interfaces [56]. The study showed that only a subset of human arachnoid granulations associates with dural sinus, yet all arachnoid granulations cores are enriched with internal cytokine and immune cells, highlighting unexplored neuroimmune properties of these structures that localize to the brain-meningeal lymphatic interface. Given the high variability in arachnoid granulations morphology and its partial associations with the dural sinus, it is suggested that arachnoid granulations form a critical interface for immune surveillance at the brain surface, thus likely to be a secondary, rather than the major site, for the extra-parenchymal CSF drainage.

These *in vivo* and post-mortem findings collectively call into question the role of arachnoid villi as a major site for CSF drainage into the bloodstream. Supporting evidence for the importance of these structures draining CSF has mostly relied on examining post-mortem tissue [45, 57]. Although human studies have reported tracer deposition in the arachnoid/dura sinus regions [58], the findings were made in aged or diseased brains where complex morphological and functional changes of arachnoid granulations likely occur [56]. Anatomically, a continuous lining of endothelial cells with tight junctions exists on the arachnoid villus, which acts as a barrier to large molecules/proteins. It is still unclear how macromolecules are transported through this barrier [59, 60]. The recent discovery of the subarachnoid lymphatic-like membrane (SLYM) - a fourth meningeal layer that separates subarachnoid space into inner and outer layers - revealed an additional barrier for the CSF solute to exit from the inner layer to the outer layer and further to the dural sinuses under normal physiological pressure [61]. These structural barriers prohibit the arachnoid villi from being an efficient site for CSF solute to enter the bloodstream, implying alternative route(s) that facilitate CSF solute into the vascular system for fast circulation.

If the arachnoid villiγsinus pathway is not a major CSF outflow route (Fig. 1d, dashed line), what would be the express entry for the solute into the bloodstream? The rapid appearance of effluxed material in venous blood draining the head (within minutes after CSF tracer injection) has been observed in multiple studies [62-64]. This timeline suggests that vasculature transport across the BBB may play a role (Fig. 1b), as the alternative paravascular CSFγlymphγbloodstream route is too slow to allow the observation of such rapid venous efflux (Fig. 1aγcγf). Clearance of exogenous substances/tracers across the BBB is mainly understudied due to the difficulty of *in-vivo* detection and quantification of tracer concentration inside the smaller brain vessels. However, BBB is widely accepted as a critical route for removing many metabolic waste solutes (e.g., amino acids, Aβ peptides) from the brain to the blood circulation through different mechanisms [13, 65]. Between the two parenchymal efflux routes, i.e., transport across BBB (Fig. 1b) and efflux along paravascular CSF (Fig. 1aγcγf), BBB is likely the express route for the waste solutes to cycle into the bloodstream.

## 4. Substance efflux across blood-brain barriers: mechanism studies

The brain's elimination of many substrates (e.g., CO_2_, glucose, lactate, amino acids, Aβ peptides) are considerably greater than could be supported by paravascular efflux alone, suggesting that BBB is an important site for solute transport [13, 66]. The BBB consists of continuous endothelial cells joined together with tight junctions which are further supported by pericytes, the basement membrane, and the astrocytic endfeet. It makes the vascular pathway in brain parenchyma highly selective in both influx and efflux of substances, as compared to the CSF pathways, due to the tight junctions in the gaps between endothelial cells [67]. The effective efflux of substances from brain parenchyma to blood (brain-to-blood) is critical for rapid transport out of the brain in large quantities to maintain brain homeostasis. Depending on the size, concentration, and chemical compositions, the brain-to-blood efflux across BBB is made through three mechanisms: via passive transfer, via transporters, and via transcytosis [13, 65].

### 4.1 Efflux via passive, non-selective transfer

The structures of BBB determine that passive, non-selective transfer can occur via a paracellular (around the endothelial cells through tight junctions) or transcellular pathway (through the endothelial cells) [68-70]. The paracellular pathway is more restrictive as blocked by the tight junctions and only allows for small solutes to transport, e.g., Na+ and Cl- [68-70]. The transcellular pathway, on the other hand, is responsible for the majority of the passively removed solutes. These solutes are either more lipid soluble (which allows them to easily diffuse across both the cell membranes and the interior of the endothelial cells) or small neutral substances such as water, methanol, ethanol, isopropanol, glycerol, ethylene glycol, urea, and thiourea [65]. The features of passive, non-selective transfer, as compared to the other two mechanisms, is that this transport does not saturate, is not inhibited by competition by other transported substances, and has no specific inhibitors. This passive permeability is a diffusion-like process that allows both influx and efflux directions [71, 72]. It has been speculated that a combination of hydrostatic and osmotic pressure gradients, known as Starling Forces, drive the cross-BBB passive transfer [73-75]. An increase in the interstitial-to-venous pressure would increase the solute efflux rate into the vein and vice versa. As a result, such efflux is likely to be affected in pathological states such as venous hypertension, where the "hydraulic push" would be reduced with the venous pressure increase.

### 4.2 Efflux via active BBB Transporters

BBB possesses virious transporters for many types of solutes located on luminal (blood-facing), abluminal (brain-facing) or both surfaces of the brain capillary endothelial cells (BCEC). ATP-binding cassette (ABC) and solute carrier (SLC) are the prominent efflux transporter families at BBB.

#### 4.2.1 ABC transporters

The discovery of ATP-binding cassette (ABC) in the early 1970s and the subsequent demonstration of their expression within the BBB added a critical element to barrier function. ABC efflux transporters belong to an ancient protein superfamily and are highly conserved among different species [76]. By consuming ATP, ABC transporters extrude metabolic wastes produced by brain parenchyma into the blood and prevent the xenobiotics from entering the brain parenchyma against the solute's concentration gradient. Detailed reviews of ABC efflux transporters can be found in [76, 77]. In the context of this review, we only focused on the transporters of ABCA, ABCB, ABCC, and ABCG families located in the membrane of the brain capillary endothelial cells (BCEC) that could efflux the substances from brain parenchyma to blood.

Transporters in ABCA family are involved in apolipoprotein (ApoE)-dependent cholesterol efflux, sterol homeostasis, and lipid metabolism. ACBA1 is the most studied ABCA transporter in CNS. Panzenboeck and colleagues [78] reported that ABCA1 is located in the abluminal membrane of BCEC. They found that ABCA1 contributed to ApoE-dependent cholesterol efflux in porcine BCEC. For other members of the ABCA family, related mRNA or protein expression has been detected in BCEC [79-82].

ABCB1 (P-glycoprotein, Pgp; multidrug resistences1, MDR1) transports amphipathic, cationic, and neutral compounds. ABCB1 was the first ABC transporter identified in a drug-resistant cell line in 1976 [83]. In 1989, Cordon-Cardo and colleagues [84, 85] found that ABCB1 existed in human brain capillary endothelial cells. The ABCB1 transporter protein is highly expressed in the luminal membrane of brain capillary endothelial cells, preventing toxins from entering the brain parenchyma. Interestingly, ABCB1 was also found in the abluminal membrane of BCEC and other locations inside the cells. However, the functions of the ABCB1 expressing in the abluminal membrane and intracellular remain unclear [86, 87]. It is worth noting that ABCB1 transports an extensive range of substrates. These substrates have large structural diversity (small molecules such as morphine, verapamil, and loperamide; peptides such as Aβ) and various classes (such as chemotherapeutics, HIV protease inhibitors, opioids, and so on) [88-96].

ABCC1 (also known as multidrug resistance protein MRP1) was first detected in the CNS in 1998 [97]. The localization of ABCC1 at the BBB is still controversial. Roberts et al. [98] reported that ABCC1 is primarily located in the abluminal membrane of BCEC, but a low level is also present in the luminal membrane. However, Cisternino S. et al. [99] found that efflux mediated by ABCC1 does not seem to occur across the luminal membrane of the BCEC. ABCC1 was reported to be associated with the efflux of 17-β-estradiol-D-17-β-glucuronide (E217βG) across BBB [100]. Besides ABCC1, other transporters in the ABCC family, such as ABCC3, ABCC4, and ABCC5, were also expressed in the membrane of BCEC. ABCC3 and ABCC5 could mediate the transport of methotrexate [101]. And ABCC4 has been demonstrated to mediate the efflux of many substances, including organic anions, glutathione-, sulfate-, or glucuronate-conjugated drugs, prostaglandins, nucleoside analogs, methotrexate, topotecan, and thiopurines [102-105].

ABCG2 (also known as breast cancer resistance protein, BCRP) was found in 1998 by Doyle et al. in a breast cancer cell line (MCF-7) that displayed high resistance to mitoxantrone [106]. In 2002, ABCG2 was detected in BBB by Eisenblätter et al. [107]. The localization of ABCG2 has been found in the luminal membrane of brain capillaries of capillary endothelial cell cultures [98, 108-112]. At BBB, ABCG2 mediates the efflux of daunorubicin, prazosin, and mitoxantrone [109, 113]. ABCG2 has also been demonstrated to efflux several exogenous chemotherapeutic drugs, such as imatinib, dasatinib, and lapatinib [109, 113-118]. Besides ABCG2, ABCG1 (White1), ABCG5 (White3, Sterolin-1), and ABCG8 (Sterolin-2) have been found on the plasma side of BCEC. They are transporters for cholesterol, sterols, and biosynthetic sterol intermediates and are responsible for cholesterol and sterol homeostasis [119].

#### 4.2.2 SLC transporters

More than 500 genes classified in 65 families named solute carrier (SLC) have been reported (see http://slc.bioparadigms.org/). Many SLC transporters have been detected in the membrane of BCEC [120]. While some SLC transporters mediate the transportation of specific substrates, others transport a relatively broad range of organic anions and cations (primarily those in the SLC21 and SLC22 families). The SLC transporters have been reviewed extensively in previous literatures [121-132]. In this review, we only focused on the SLC transporters that could efflux the substances from brain parenchyma to blood. The SLC transporters reviewed in this review included SLC1, SLC6, SLC7, SLC16, SLC21, SLC22, SLC38.

Members of the SLC1 family are glutamate transporters [133]. SLC1A3 (GLAST), SLC1A2 (GLT1), and SLC1A1 (EAAC1) have been detected in the abluminal membrane of bovine brain microvessels and been found to catalyze glutamate efflux from the brain parenchyma [134, 135]. SLC1A5 localized at the abluminal side of BCEC mediates the L-aspartate efflux from the parenchyma [136].

SLC6 is the sodium- and chloride-dependent neurotransmitter transporter family. SLC6A6 (TAUT) has been detected in the rat BCEC [137], which mediates the transport of taurine (2-amino-ethane sulfonic acid). SLC6A13 (GAT2/BGT-1) has been detected in the cultured brain endothelial cells. It mediates the removal of GABA and betaine from the brain parenchyma [138]. SLC6A2 (NET) and SLC6A4 (SERT) have been detected in mouse brain capillary cell line, which mediates the transport of norepinephrine and serotonin, respectively [139].

The SLC7 family mediates the transportation of amino acids. It can be divided into two subfamilies: the cationic amino acid transporters (CATs) and L-type amino acid transporters (LATs). SLC7A5 (LAT1) is a glycoprotein-associated transporter belonging to LATs. Boado and colleagues [140] showed that SLC7A5 mediates the transport of amino acids through the BBB. SLC7A11 (also known as xCT) is a cystine/glutamate antiporter that belongs to LATs. L-Aspartate and L-glutamate are the predominant substrates of SLC7A11 [141]. It has been reported that SLC7A11 expresses in the luminal membrane and mediates the efflux of glutamate into the blood [142].

The SLC16 family has been first detected in the 1970s as a specific L-lactate membrane transporter [143]. SLC16A1 (MCT1) is the first cloned member of its family, which mediates the transportation of short-chain monocarboxylates [144, 145]. Gerhart et al. [146] found SLC16A1 in rat brain endothelium's luminal and abluminal membranes. Besides nutrient transport, MCT1 is also responsible for removing lactate from the brain parenchyma to endothelial cells and plasma, especially under pathological conditions [28].

The SLC21 family mediates the transport of organic anions. In this family, SLCO1A4 (OATP2) has been detected in the rat brain and mouse BCEC [147]. The localization of SLCO1A4 is postulated to be in the luminal and abluminal membranes of the BCEC [148]. It mediates the efflux of various substrates from the brain parenchyma. The substrates of SLCO1A4 include glucuronides, sulfates, glutathione conjugates, δ-opioid receptor agonists, and valproic acid [149-151].

SLC22A5 (OCTN2) is an organic cation transporter in the SLC22 family. It has been detected in the primary culture of the BCEC and the cell lines [152-156]. The localization of SLC22A5 was postulated to be in both the luminal and abluminal sides of the BBB. The substrates of SLC22A5 are several organic cations, such as β-lactam antibiotics [157, 158].

SLC38 is a family of neutral amino acid transporters. SLC38A3 (SNAT3) has been detected in the mouse capillary luminal and abluminal membranes, which mediates glutamine transport in both directions. SLC38A2 (ATA) was seen in the abluminal side of rat BBB. It mediates the efflux of small amino acids, including L-proline, glycine, and L-alanine [159].

In summary, substrate efflux via transporters located on the membranes of BCEC has been extensively investigated. Ample evidence demonstrated that these transporters are a critical pathway for waste clearance from the parenchyma, as substrate efflux through SLC has revealed a clearance volume much greater than the clearance associated with the paravascular route (Table 1 in [65]). The evidence further showed that these transporters are responsible for the quick elimination of solute from the parenchyma, resulting in the rapid appearance of effluxed material in venous blood draining the head.

### 4.3 Efflux via transcytosis

Transcytosis is an active transcellular efflux mechanism for the transport of large substrates across the BBB through either absorptive-mediated transcytosis (AMT) or receptor-mediated transcytosis (RMT). AMT usually starts with the adsorption of substrates onto the surface of caveolae, while RMT is initialized by binding substrates with specific receptors. These initialization events lead to endocytosis flowed by delivery of the vesicles to the cell membrane of the opposite side [160-162]. There is evidence that transcytosis can occur in either direction, i.e., from the brain to blood and from blood to brain. The large substrates that are transported from brain to blood through the transcytosis pathway include Aβ peptides (via low-density lipoprotein receptor-related protein LRP1 and LRP2) [163, 164], insulin (via insulin receptor), [165, 166], transferrin (via the transferrin receptor, TfR) [167, 168], and IgG molecules (via interaction with an unidentified receptor). Note that while both BBB, paravascular, and periarterial routes have been identified for Aβ efflux, studies have shown that LRP1-dependent transcytosis plays a substantial role in Aβ elimination, with LRP1 knocked out mice showing 48% reduction in Aβ removal [169, 170]. The first glymphatic study also reported a much faster Aβ clearance than mannitol or dextran, where the latter two substances lack specific efflux receptors, supporting receptor-mediated efflux of Aβ across the BBB [6].

Collectively, these well-studied mechanisms demonstrate the ability of cerebral vasculature to drain metabolite waste substance across blood-brain barriers in large quantities. Besides brain's innate waste substance, many "blood-to-brain impermeable" tracers have been observed to drain into the cerebral veins following cisterna-magna tracer injections [25, 26, 171]. While the exact mechanism for their brain-to-blood transport across BBB awaits further investigation, these observations suggest a role of vascular participation in draining exogenous tracers, as discussed in detail below.

## 5. Parenchymal vasculature participates in substance clearance: Imaging and immunohistochemical studies

The strongest evidence for vascular participation in waste clearance comes from MRI and immunohistochemical studies. In this section, we will discuss the MRI technique and review the evidence for the direct involvement of parenchymal vasculature in substance clearance.

### 5.1 MRI *vs.* two-photon microscopy imaging technique

As a hydraulic system, both the paravascular and vascular spaces must be studied intact without any leakage, ideally with minimal perturbation to the system and ultra-high detection sensitivity. Two-photon microscopy and MRI are the most used imaging techniques for studying brain waste clearance. Two-photo microscopy has ultra-high detection sensitivity and has made ground-breaking discoveries, including the glymphatic system and solving the long-time puzzle of why we need sleep [172]. However, due to technical limitations, it can only image the brain surface with a limited penetration depth (i.e., 250µm). Therefore, it can only capture regional/partial paravascular and vascular pathways. As a result, two-photon imaging may miss some critical influx/efflux routes that prevent it from answering whether parenchymal vasculature directly participates in substance clearance. In comparison, the MRI-based superparamagnetic iron oxide enhanced susceptibility weighted imaging (SPIO-SWI) has a whole-brain coverage. It can overcome insufficient spatial resolution in conventional MRI with a marked increase in detection sensitivity. Therefore, SPIO-SWI may provide a more comprehensive picture of influx and efflux pathways.

### 5.2 The SPIO-SWI technique

Conventional MRI cannot simultaneously study the micro-vessels and their surrounding paravascular space due to limited imaging resolution and the small sizes of these spaces. SWI overcomes the limited imaging resolution by incorporating the phase information of the image. MRI provides both magnitude and phase information [173, 174], yet the phase information is usually discarded in conventional MRI. The susceptibility-induced phase signal can amplify the underlying signal source and boost the detection sensitivity. Combined with the superparamagnetic iron oxide (SPIO), SPIO-SWI can offer a blooming effect that can significantly increase the detection sensitivity of sub-pixel micro-vessels, providing the capability to distinguish between paravascular and adjacent intra-vascular spaces.

It has been demonstrated SPIO-SWI can increase the MRI-measured vessel diameter by 13.3-fold and 19.9-fold with 5.6 mg Fe/kg and 16.8 mg Fe/kg ferumoxytol [175]. Moreover, SPIO-SWI provides excellent contrast between CSF (dark signal due to SPIO) and blood (bright signal due to inflow effect). Therefore, SPIO-SWI becomes a superb tool for studying the clearance pathways along both paravascular and vascular networks simultaneously given its whole-brain coverage, high detection sensitivity, and enhanced contrast between blood and CSF.

### 5.3 MRI evidence for the direct participation of parenchymal vasculature in substance clearance with the intrathecal administration of CSF tracers

As discussed, imaging the intrathecally administered CSF tracers can reveal clearance pathways due to the unique CSF network. Combined with the SPIO-SWI technique, determining whether parenchymal vasculature directly participates in substance clearance becomes simple. If tracers in venous blood flowing out of the brain parenchyma are more than that in the arterial inflow, we can conclude that tracers in the brain parenchyma enter venous blood [26].

Three experiments were conducted with observations that unanimously support the direct participation of parenchymal vasculature in substance efflux. For the first experiment, the signal intensity profile at the vein and artery and corresponding para-venous and para-arterial space was qualitatively estimated before and after the intra-cisterna magna infusion of 100μg Fe-dextran (100nm). The decrease of signal intensity from both para-arterial and para-venous space indicated the MRI tracers transported through the paravascular space, consistent with the glymphatic theory. More importantly, while the signal intensity from the artery (azygos pericallosal artery) remained unchanged, the signal intensity from the veins (azygos internal cerebral vein) decreased. This indicated that MRI tracers enter the vein but not the artery, suggesting the participation of parenchymal veins in draining the Fe-dextran tracer.

For the second experiment, CSF tracers were quantitatively measured in both the vein and artery using a similar experimental setup but with a 75 μg (Fe) FeREX™ (50-150 nm) tracer. The relative signal intensity changes in the veins decreased by 16.0 (± 4.1) % at 15 min post-tracer infusion compared to baseline (pre-tracer) (p < 0.01). In contrast, the relative signal changes in the arteries were not significantly different (p >0.01) [26]. In other words, the outflow of tracers in venous blood was more than its inflow in arterial blood, suggesting its drainage through the veins.

For the third experiment, the susceptibility of the MRI tracers in both the vein and artery was estimated by quantitative susceptibility mapping (QSM) to reduce possible blooming effects on arteries and veins. Greater QSM changes in the vein than in the artery were detected at both post-15min and post-45min 75μg FeREX™ (100nm) CSF injection, suggesting more tracers in the vein than in the artery. Moreover, the comparison between MRI integral signal in the vein and adjacent para-venous space gave a ratio of 32:100, suggesting a non-ignorable contribution of venous efflux in tracer clearance, in addition to the para-venous drainage.

### 5.4 Immunofluorescence study indicated the presence of CSF tracer in the BBB

Immunofluorescence imaging was used to study brain tissue two hours after injecting FITC-dextran (molecular weight 10KDa) tracer into the right ventricle. The presence of CSF tracer within the endothelial walls of capillaries and small vessels was observed [25]. An analogous result was reported by Wagner et al [176], where they identified vesicular transport within both vacular endothelial cells and perivascular microglia as primary mechanisms for clearing horseradish peroxidase from CSF into blood plasma. These histology findings echo with the in vivo imaging results [26], which collectively support the vascular involvement in parenchymal substance clearance.

## 6. Implications for understanding neuro-degenerative diseases

As discussed, substance clearance in the brain parenchyma relies on both the vascular system and the glymphatic system. Impairments of either system have been observed in neurodegenerative diseases such as AD [9, 67, 177-190], Parkinson’s disease [191-201], and traumatic brain injury [202-208] in both animal and human studies. For example, damages to cerebro-vasculature, such as the increased vascular permeability or transporter dysfunction across BBB, were associated with AD pathologies (Aβ and tau) and contributed to the onset and progression of AD [67, 177-183]. And impaired glymphatic system has also been found in AD pathogenesis [9, 184-190]. These studies suggested that integrity of both vascular and glymphatic functions are critical to brain waste clearance. Dysfunction of either one may cause waste buildup and trigger neurodegenerative diseases [209].

Therefore, simultaneous evaulation of cerebro-vascular and glymphatic functions can be beneficial in studying neurodegenerative diseases. Current imaging techniques mainly evaluate cerebrovascular or glymphatic function alone, e.g., targeting either perfusion or CSF [14, 178, 189, 194, 205, 209-213]. SPIO-SWI holds promises in simultaneously evaluating waste drainage along both vascular and glymphatic pathways with clinical translation potential [26, 171]. Also, multimodal imaging techniques could be incorporated to simultaneously assess both clearance pathways to gain a complete view of brain’s clearance function.

Therapeutic strategies that target both vascular and glymphatic systems rather than target either system alone could be explored to achieve better treatment effects. Drugs and intervention strategies targeting either cerebrovascular or glymphatic dysfunctions have been investigated to prevent or alleviate neurodegenerative diseases [214-221]. Yet, the impairments of cerebrovascular and glymphatic function may share common mechanisms. For example, the deficiency of NOTCH3, primarily expressed in vascular smooth muscle cells (vSMCs), could impair cerebral vessel architecture, contractility, and glymphatic function [222]. And disrupted glymphatic function has been observed when cardiovascular functions were altered [15, 16, 18, 223]. Therefore, considering both clearance systems during the development of future therapies could prove to be advantageous.

While our focus is the brain parenchyma, we could speculate that this dual clearance system—the combined vascular and CSF clearance—may apply to waste clearance function in other parts of the central nervous system, including the spinal cord and the neural parts of the eye and inner ear. Dysfunctional water drainage in the ocular and the inner ear may underlie glaucoma and Meniere disease, both of which are characterized by an excess of local water [224]. Recent studies have suggested an ocular glymphatic system based on the observance of Aβ clearance along the optic nerves and intravenously injected GBCA leaking into the CSF surrounding optic nerves [225-227]. The studies further proposed that glymphatic dysfunction may underlie the water accumulation. Based on our previous discussion, we suspect altered vasculature and CSF drainage may contribute to the excess water. Thus, both pathways are worth investigating in future studies of these diseases.

## 7. Conclusion

Together, the evidence from different perspectives highlights a contribution of the cerebral vascular system in brain clearance: 1) the intra-parenchymal vascular transport may be the express entry for the solute into the bloodstream that underlies the rapid appearance of effluxed material in venous blood observed in many studies, especially given that the arachnoid villi/granulations as the main site for solute entering the vascular system have been questioned; 2) multiple blood-brain barrier transport mechanisms have been identified for the parenchymal vascular drainage; 3) recent MRI studies of CSF tracer efflux pathways support direct participation of the parenchymal venous system in draining the intra-cisterna magna infused tracers, complimenting the paravascular CSF drainage (glymphatic system); Furthermore, the different sizes of CSF tracers can enter the parenchymal venous system but not arterial vessels, reflecting a directional transport of BBB. While the relative contribution of vascular and CSF pathways in waste clearance is yet to be studied and maybe substance dependent, the parenchymal vascular drainage is logically consistent with the notion that the venous system outside the brain drains about 90% of the interstitial fluid, with the remaining 10% drained through the lymphatic system that has the similar circulation speed as the CSF [228]. As the brain is the most bioactive, energy-consuming organ, it is logical that vascular clearance plays a role, particularly considering less bioactive peripheral tissues require both the fast vascular and slow lymphatic systems to remove substances efficiently. In conclusion, the parenchymal vascular contribution to the brain's substance clearance should gain more attention in future studies. Understanding the brain’s reliance on the two pathways is crucial for unveiling the underlying mechanism of neurodegenerative diseases and for developing effective diagnostic and therapeutic approaches.
